# Sertoli and Germ Cells Within Atrophic Seminiferous Tubules of Men With Non-Obstructive Azoospermia

**DOI:** 10.3389/fendo.2022.825904

**Published:** 2022-06-02

**Authors:** Christian Fuglesang Skjødt Jensen, Danyang Wang, Linn Salto Mamsen, Aleksander Giwercman, Niels Jørgensen, Mikkel Fode, Dana Ohl, Lihua Dong, Simone Engmann Hildorf, Susanne Elisabeth Pors, Jens Fedder, Elissavet Ntemou, Claus Yding Andersen, Jens Sønksen

**Affiliations:** ^1^ Department of Urology, Herlev and Gentofte Hospital, Herlev, Denmark; ^2^ Department of Clinical Medicine, University of Copenhagen, Copenhagen, Denmark; ^3^ Laboratory of Reproductive Biology, University Hospital of Copenhagen, Rigshospitalet, Copenhagen, Denmark; ^4^ Department of Translational Medicine and Reproductive Medicine Centre, Lunds University and Skane University Hospital, Malmö, Sweden; ^5^ Department of Growth and Reproduction, University Hospital of Copenhagen, Rigshospitalet, Copenhagen, Denmark; ^6^ Department of Urology, University of Michigan, Ann Arbor, MI, United States; ^7^ Centre of Andrology & Fertility Clinic, Department D, Odense University Hospital, Odense C, Denmark

**Keywords:** non-obstructive azoospermia, un-dilated tubules, hypospermatogenesis, maturation arrest, Sertoli cell only, male infertility

## Abstract

**Background:**

Infertile men with non-obstructive azoospermia (NOA) have impaired spermatogenesis. Dilated and un-dilated atrophic seminiferous tubules are often present in the testes of these patients, with the highest likelihood of active spermatogenesis in the dilated tubules. Little is known about the un-dilated tubules, which in NOA patients constitute the majority. To advance therapeutic strategies for men with NOA who fail surgical sperm retrieval we aimed to characterize the spermatogonial stem cell microenvironment in atrophic un-dilated tubules.

**Methods:**

Testis biopsies approximately 3x3x3 mm^3^ were obtained from un-dilated areas from 34 patients. They were classified as hypospermatogenesis (HS) (n=5), maturation arrest (MA) (n=14), and Sertoli cell only (SCO) (n= 15). Testis samples from five fertile men were included as controls. Biopsies were used for histological analysis, RT-PCR analysis and immunofluorescence of germ and Sertoli cell markers.

**Results:**

Anti-Müllerian hormone mRNA and protein expression was increased in un-dilated tubules in all three NOA subtypes, compared to the control, showing an immature state of Sertoli cells (p<0.05). The GDNF mRNA expression was significantly increased in MA (*P*=0.0003). The BMP4 mRNA expression showed a significant increase in HS, MA, and SCO (*P*=0.02, *P*=0.0005, *P*=0.02, respectively). The thickness of the tubule wall was increased 2.2-fold in the SCO-NOA compared to the control (p<0.05). In germ cells, we found the DEAD-box helicase 4 (DDX4) and melanoma-associated antigen A4 (MAGE-A4) mRNA and protein expression reduced in NOA (MAGE-A: 46% decrease in HS, 53% decrease in MA, absent in SCO). In HS-NOA, the number of androgen receptor positive Sertoli cells was reduced 30% with a similar pattern in mRNA expression. The γH2AX expression was increased in SCO as compared to HS and MA. However, none of these differences reached statistical significance probably due to low number of samples.

**Conclusions:**

Sertoli cells were shown to be immature in un-dilated tubules of three NOA subtypes. The increased DNA damage in Sertoli cells and thicker tubule wall in SCO suggested a different mechanism for the absence of spermatogenesis from SCO to HS and MA. These results expand insight into the differences in un-dilated tubules from the different types of NOA patients.

## Introduction

Azoospermia is identified in 1% of all men and up to 20% of infertile men, and is subdivided as obstructive and non-obstructive azoospermia (NOA) (1, 2). NOA, the most severe form of impaired male fertility, is the absence of spermatozoa in the ejaculate caused by reduced or missing spermatogenesis (3). Several potential causes of NOA are known, including varicocele, previous gonadotoxic therapy, Y chromosome microdeletions and Klinefelter’s Syndrome, but in up to half of the cases a clear etiology is not identified (4). Based on the histological assessment of testis biopsies, men with NOA, independent of cause, can be classified into one of the three categories: Hypospermatogenesis (HS) - decreased spermatogenesis with all types of germ cells present, maturation arrest (MA) - premature arrest of spermatogenesis, and Sertoli cell-only (SCO) - absence of germ cells (5, 6).

In men diagnosed with NOA, surgical retrieval of spermatozoa, which can be used for intracytoplasmic sperm injection (ICSI), is performed. However, sperm retrieval rates are approximately 50% (7), leaving half of all NOA patients to rely on donor sperm. Novel therapeutic techniques, such as spermatogonial stem cell-based transplantation or autologous tissue transplantation, might represent alternatives to restore fertility in the future (8, 9). In order to advance these strategies more detailed information on the microenvironment of the seminiferous tubules including germ cells and Sertoli cells from different types of NOA patients is required.

Sertoli cells in the seminiferous tubules support the niche in which the spermatogonial stem cells (SSCs) differentiate into spermatozoa (10, 11). A delicate and intricate hormonal balance between Sertoli cell function and SSCs is required to support full spermatogenesis, which also involves Leydig cells providing sufficient amounts of androgens. Testosterone from Leydig cells acts *via* the androgen receptor (AR) which is expressed in mature Sertoli cells but not in the germ cells. Moreover, Anti-Müllerian hormone (AMH) is secreted by immature Sertoli cells and is downregulated by increased intratesticular testosterone during puberty (12). Sertoli cells produce glial cell-derived neurotrophic factor (GDNF) and bone morphogenetic protein 4 (BMP4) that influence undifferentiated spermatogonia in rodent but their role remains unclear in human (13–16). Peritubular myoid cells (PTMCs) constitute the tubular wall and act to contract seminiferous tubules leading to the transfer of immotile sperm. PTMCs exerts paracrine functions on Sertoli cells and Leydig cells (17, 18). Activations of Leydig cell expressed luteinizing hormone(LH) receptor lead to production of testosterone which signal through AR receptors expressed on Sertoli cells (19). Collectively, successful spermatogenesis is dependent on proper function of several cell types in the testis and hormonal stimulation, which in NOA patients are aberrant in one or more, currently unknown, steps.

Men with NOA occasionally have pockets of “dilated” seminiferous tubules. These tubules are likely to have a normal diameter but appear dilated in relation to the surrounding atrophic “un-dilated” tubules. For practical reasons the terms “dilated” and “un-dilated” will be used in the remainder of the text. Dilated tubules often manage, for unknown reasons, to sustain active spermatogenesis while the un-dilated areas lack this ability. Extraction of sperm cells from the dilated tubules by microdissection testicular sperm extraction (mTESE) is used clinically to obtain spermatozoa for ICSI. In an attempt to advance understanding of reasons for the spermatogenic impairment in the un-dilated seminiferous tubules in NOA patients, we explored different markers of germ and Sertoli cells using mRNA expression and immunofluorescence. Thus, in order to understand how to advance spermatogenesis in testis tissue from NOA patients, we aimed at understanding the difference in molecular characteristics of germ cells and Sertoli cells between testes from men with normal spermatogenesis and atrophic un-dilated tubules in testis biopsies from NOA men with HS, MA and SCO.

## Materials and Methods

### Human Testis Materials

Testis tissue was obtained from 34 NOA patients who underwent mTESE as a part of treatment for infertility and from five adult men with normal sperm production and proven fertility, who provided a biopsy in connection with vasectomy. In patients with NOA, testis biopsies approximately 3x3x3 mm^3^ were obtained anteriorly right under the tunica albuginea in connection with the mTESE procedure and used for histopathological diagnosis. Additional testis biopsies that did not show dilated seminiferous tubules (as observed under the operating microscope) were taken from the same area. Consequently, in NOA men, only biopsies from un-dilated tubules were used in this study. All testicular biopsies for research purposes were placed immediately in McCoy 5A medium (modified 22330-021, Gibco, UK) for transportation to the Laboratory of Reproductive Biology for the cryopreservation. Then they were equilibrated for 20 min in media consisting of 1.5 M ethylene glycol, 0.1 M sucrose, 10 mg/ml human serum albumin (HSA) (CSL Behring, Germany), frozen and cryopreserved in -196°C liquid nitrogen according to previous published methods (20).

### Clinical Workup

All men with NOA were diagnosed after a complete medical history and physical examination including scrotal ultrasound. Azoospermia was diagnosed according to the 5^th^ edition of World Health Organization (WHO) laboratory manual for the “Examination and processing of human semen” (21). A full hormonal evaluation including serum levels of follicle-stimulating hormone (FSH), LH, inhibin. B and testosterone was performed. All men were assessed for the presence of Y chromosome microdeletions and a karyotype was obtained. Fasting morning blood samples were drawn. Serum testosterone levels were analyzed by a chemiluminescence immunoassay (Access 2, Beckman Coulter, Brea, CA, USA), follicle-stimulating hormone FSH and LH by a time-resolved immunofluorometric assay (Delfia, Wallac, Turku, Finland), and inhibin B by a specific two-sided enzyme-immunometric assay (Inhibin B gen II, Beckman Coulter Ltd, High Wycombe, UK). Culture of peripheral blood lymphocytes was used for karyotype analysis. The diagnosis of NOA was made after a complete assessment by an experienced andrologist using all the above information. Men with testis size larger than 15ml, indication of obstructive causes of azoospermia and Klinefelter’s Syndrome were not included.

### Tissue Processing and Histology

Thawing was done by progressively using the following three thawing media. Thawing medium I: 0.75 M ethylene glycol, 0.25 M sucrose in PBS, and 10 mg/ml HSA; thawing medium II: 0.25 M sucrose in PBS, and 10 mg/ml HSA; thawing medium III: PBS and 10 mg/ml HSA, each medium for 10 min (20). After thawing, one testis biopsy from each patient was divided into three parts. One part for immunostaining, one for qPCR, and one was re-frozen for future use. Tissues for immunostaining were fixed in 4% paraformaldehyde (PFA) at room temperature overnight, embedded in paraffin and cut in 5-μm sections. Sections were deparaffinized in xylene, rehydrated with series of graded ethanol. Sections for histological evaluation were stained with periodic acid-Schiff reagent (PAS). Due to the heterogeneity of testis tissues in men with NOA, the spermatogenetic status of all 34 samples was histologically re-analyzed on sections stained with PAS in addition to the original histopathological diagnosis made as part of clinical care using a 3x3x3 mm^3^ biopsy taken anteriorly under the tunica albuginea. If different, the histopathological diagnosis from un-dilated tubules was used.

### Immunofluorescence Staining

After deparaffinization and rehydration of the section, antigens were retrieved by boiling in TEG buffer (10 mM Tris, 0.5 mM ethylene glycol-bis (2-aminoethylehter)-N, N, N’, N’-tetraacetic acid (EGTA), pH 9) for 30 min. After non-specific binding was blocked with 1% bovine serum albumin (BSA) in Tris-buffered saline (TBS) buffer (50 mM Tris, 150 mM NaCl, pH7.6) for 30 min, the sections were incubated with primary antibodies at +4°C overnight. All antibodies were diluted in TBS with 1% BSA. The primary antibodies included (**Supplementary Table 1**): a monoclonal mouse anti-melanoma antigen genes-A (MAGE-A) (1:100) for detection of MAGE-A1, MAGE-A2, MAGE-A3, MAGE-A4, MAGE-A6, MAGE-A10, MAGE-A12 which were previously shown to be present in spermatogonia and some spermatocytes (22, 23), a monoclonal mouse anti-ubiquitin carboxyl-terminal hydrolase L1 (UCHL1, also known as protein gene product 9.5, PGP 9.5) (1:100) present in human spermatogonia (24), a polyclonal rabbit anti- phosphorylation of histone H2AX in Serine 139 (γH2AX) (meiotic marker in germ cells and a marker of DNA double-strand breaks in somatic cells) (1:1500) (25, 26), a monoclonal mouse anti-Vimentin (1:200) for somatic cells (27), a polyclonal rabbit anti-SOX9 (1:100) for Sertoli cells (28), a polyclonal goat anti-Müllerian hormone (AMH) (1:100) for immature Sertoli cells (28), a monoclonal rabbit anti-androgen receptor (AR) (1:100) for mature Sertoli cells (29), a rabbit polyclonal anti-alpha-smooth muscle actin (ACTA) (1:150) for peritubular myoid cells (30), a goat polyclonal anti-cytochrome P450 17A1 (CYP17A1) (1:200) for Leydig cells (23, 31). And universal negative control serum (NC498H, Biocare Medical) was used for negative control. After washing 3 times in TBS with Tween 20^®^, the slides were incubated with the following secondary antibodies at room temperature for 1h: FITC-conjugated donkey anti-mouse IgG antibody/Alexa Fluor 594 donkey anti-rabbit IgG antibody/Alexa Fluor 568 donkey anti-goat IgG antibody (1:500, Jackson ImmunoResearch). After washing, the slides were stained with 4’,6 – diamidino-2-phenylindole (DAPI) for nuclear staining. Pictures were taken on a Zeiss Axiophot microscope, operated with a Leica DFC420C digital microscope camera and LAS V4.9 software (Leica).

Five seminiferous tubules per section were randomly chosen (two tubules from upper panel, two tubules from lower panel, one tubule from the center) and two histological sections at different depths of the biopsy were evaluated per sample. The number of SOX9/AR-positive Sertoli cells and MAGE-A-positive germ cells per square millimeter (mm^2^) was calculated. Firstly, we measured the diameter of the tubule to calculate the area (mm^2^) of the tubule. Then, we counted the number of SOX9/AR/MAGE-A-positive cells within each tubule. We got the number of SOX9/AR/MAGE-A-positive cells/tubule area (mm^2^). Finally, we calculated the mean number of SOX9/AR/MAGE-A-positive cells/mm^2^ based on 10 tubules per testis biopsy. For the MAGE-A-positive germ cell, we only counted positive cells located on the basement membrane to exclude counting of spermatocytes. We evaluated AMH expression based on its staining intensity from “strong” “moderate” “mild” to “absent”. The thickness of tubule wall was measured at four points around the circumference of each tubule, with ACTA-positive signal thickness measured at the ends of two perpendicular axes.

### RNA Extraction and Quantitative RT-PCR

RNA was extracted from testis biopsies from all 34 NOA patients and five normal control testis tissues using Trizol reagent (Invitrogen) and 1-bromo-3-chloropropane (Sigma). Then the following steps were performed using the RNeasy Kit (Qiagen) according to the manufacturer’s protocol. The average RNA obtained was 22ug with a range from 5ug to 80ug. The 260/280 ratio was found to be 2.1 ± 0.04 (range: 2.00 to 2.14). The total RNA from each sample used to make the cDNA was 1ug. cDNA was synthesized by using of High-Capacity cDNA Reverse Transcription Kit (Applied Biosystems). The dilution of the cDNA template used to do qPCR was 1:5.

Quantitative RT-PCR was performed by using TaqMan Fast Advanced Master Mix (Applied Biosystems) and the LightCycler 480 Instrument II (Roche Diagnostics). The cDNA from each sample was used as a RT-PCR template to be detected of the expression of those genes from TaqMan primer assays (**Supplementary Table 2**). GAPDH was used as an internal control and the normal adult testis tissue as a positive control.

### Statistics

Individual values were presented as mean ± standard deviation (sd). GraphPad Prism version 8.0 was used for statistical analyses. Kruskal-Wallis test with Dunn’s multiple comparisons test was used to analyze the difference of mRNA expression among HS, MA, SCO to the normal control group, respectively. For the histological results, each subtype of NOA was compared to the normal control. The statistical difference of the number of MAGE-A/SOX9/AR-positive cells and the thickness of tubule wall among HS, MA, SCO to the normal control group was analyzed by Kruskal-Wallis test with Dunn’s multiple comparisons test. Chi-square test was used to analyze the percentage of AMH-positive tubules in NOA subtypes compared to normal control group. Correlations between mRNA expression and hormone levels were tested by Spearman rank correlation coefficient. *P* values < 0.05 were considered statistically significant.

## Results

### PAS Staining for Histopathological Diagnosis

Based on histological evaluation of biopsies from the un-dilated areas of seminiferous tubules and the clinical diagnosis, our material consisted of 5 HS, 14 MA, and 15 SCO samples (**Table 1**).

**Table 1:** Age and etiology of non-obstructive azoospermia (n = 34) and normal control.

Variable	HS (n = 5)	MA (n = 14)	SCO (n = 15)	Normal control (n = 5)
Age years, mean (sd)	33 (3)	32 (6)	35 (6)	32 (5)
Cryptorchidism N (%)	2 (40.0)	5 (35.7)	4 (26.7)	–
Varicocele N (%)	3 (60.0)	5 (35.7)	5 (33.3)	–
Idiopathic N (%)	–	3 (21.4)	5 (33.3)	–
AZFc N (%)	–	1 (7.1)	–	–
Cancer treatment N (%)	–	–	1 (6.7)^a^	–

a This patient had a 46, X, inv (Y) (p11.2 q11.22) karyotype, all other included NOA patients all had normal karyotype.

### Characterization of Niche Related Cells in the Testis From Different Types of NOA Patients Using Immunofluorescence Staining

#### Somatic Cells

**Figure 1:**
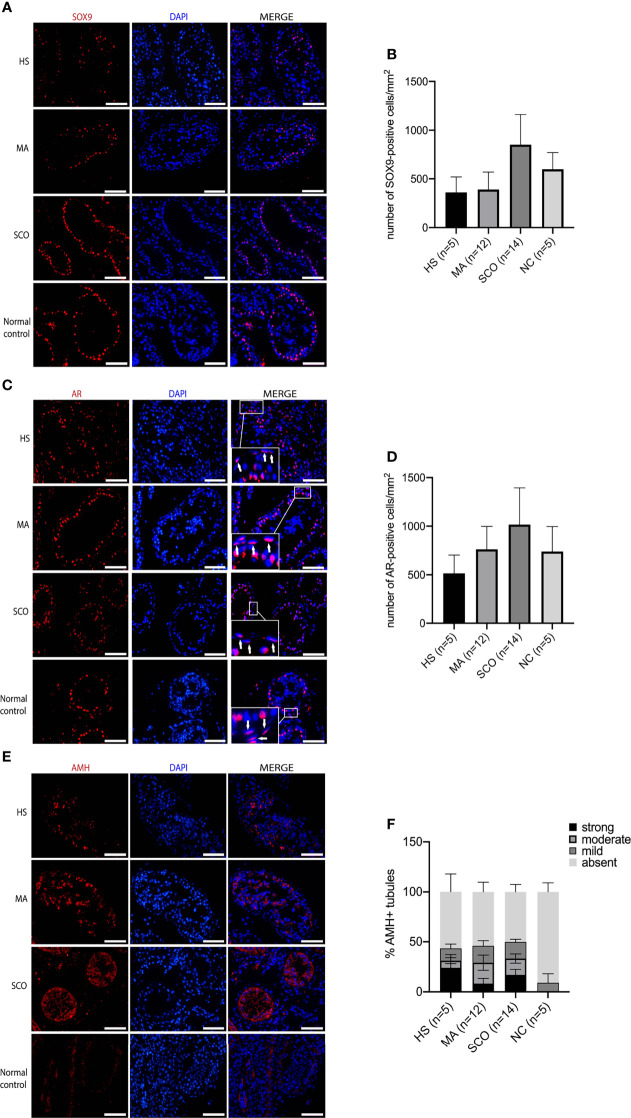
Immunofluorescence staining of Sertoli cell markers in un-dilated seminiferous tubules from hypospermatogenesis (HS), maturation arrest (MA), Sertoli cell only (SCO), and normal control (NC) samples. **(A)** Sox9 (red) for Sertoli cells. **(B)** The number of Sox9-positive cells per mm^2^ was counted from ten tubules of each biopsy. **(C)** AR (red) for mature Sertoli cells, white arrow indicated that AR also expressed in PTMCs. **(D)** AR-positive cells per mm^2^ was counted from ten tubules of each biopsy. **(E)** AMH (red) for immature Sertoli cells. **(F)** AMH staining was categorized into “strong” “moderate” “mild” “absent” based on staining intensity. The percentage of AMH-positive tubules (including strong, moderate, mild expression of AMH) showed significant increase in HS, MA, and SCO comparing to normal control group. The percentage of tubules with “strong” AMH expression in each NOA subtypes was significantly increased to normal control (HS: *P* < 0.001, MA: *P* = 0.001, SCO: *P <* 0.001). The percentage of tubules with “moderate” AMH expression in each NOA subtypes was also significantly increased (HS, MA, SCO: *P*< 0.001). The percentage of “mild” AMH expression only significantly increased in SCO (*P* = 0.006). DAPI (blue) for nuclear staining. Scale bar: 100 μm. n represent the number of individuals included.

SOX9-positive Sertoli cells appeared disorganized with a scattered distribution in HS while organized with a more circle like location close to the basal membrane in MA compared to normal group, contrasting the SCO samples which resembled the normal tissue with cells tidily located close to the basement membrane and near one another to each other (**Figures 1A, B** **)**. The total number of SOX9-positive cells was counted in ten tubules and the average number per tubule was 20 in HS, 19 in MA, 24 in SCO, and 18 in normal control group. The average number of AR-positive cells per tubule was 21 in HS, 22 in MA, 24 in SCO, and 28 in normal control group. Combining the different size of tubules, the number of SOX9/AR-positive Sertoli cells per mm^2^ was not significantly different in NOA subtypes compared to the normal group (**Figures 1A-D**). The percentage of AMH-positive tubules showed significant increase in HS, MA, and SCO compared to normal control group (HS, MA, SCO: *P*<0.001). The percentage of tubules with “strong” AMH expression in each NOA subtypes was significantly increased to normal control (HS: *P*<0.001, MA: *P*=0.001, SCO: *P*<0.001). The same significant increase was observed in tubules with “moderate” AMH expression (HS, MA, SCO: *P*<0.001). The percentage of tubules with “mild” AMH expression significantly increased only in SCO (*P*=0.006) (**Figures 1E, F** **)**.

**Figure 2:**
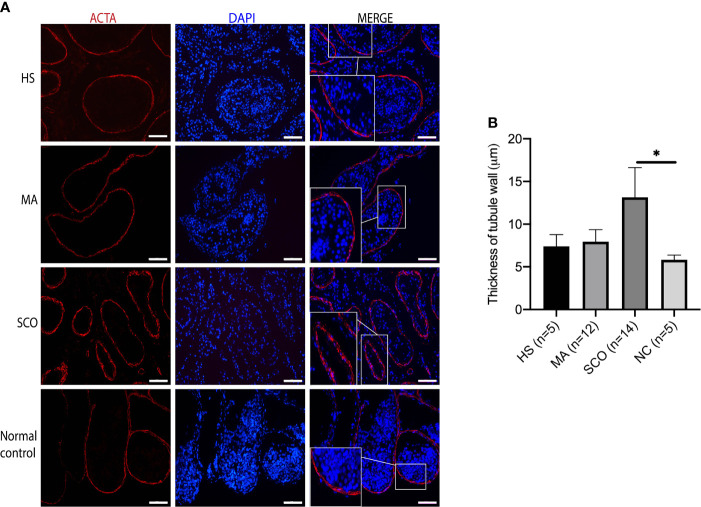
Immunofluorescence staining of peritubular myoid cell (PTMC) marker in un-dilated seminiferous tubules from HS, MA, SCO, and NC samples. **(A)** Alpha-smooth muscle actin (ACTA) (red) for PTMC, DAPI (blue) for nuclear staining, Scale bar: 100 μm. **(B)** Thickness analysis of tubule wall. Asterisk indicated significant difference between SCO and normal control group (*p < 0.05).

Visualizing the peritubular myoid cells (PTMCs) *via* ACTA-positive staining, the distribution of cells and the thickness of tubule wall appeared to be normal in both HS and MA patients, while the tubule wall in the SCO patient was 2.2-fold thicker than in the normal testis tissues (*P* = 0.001) (**Figures 2A, B** **)**.

**Figure 3:**
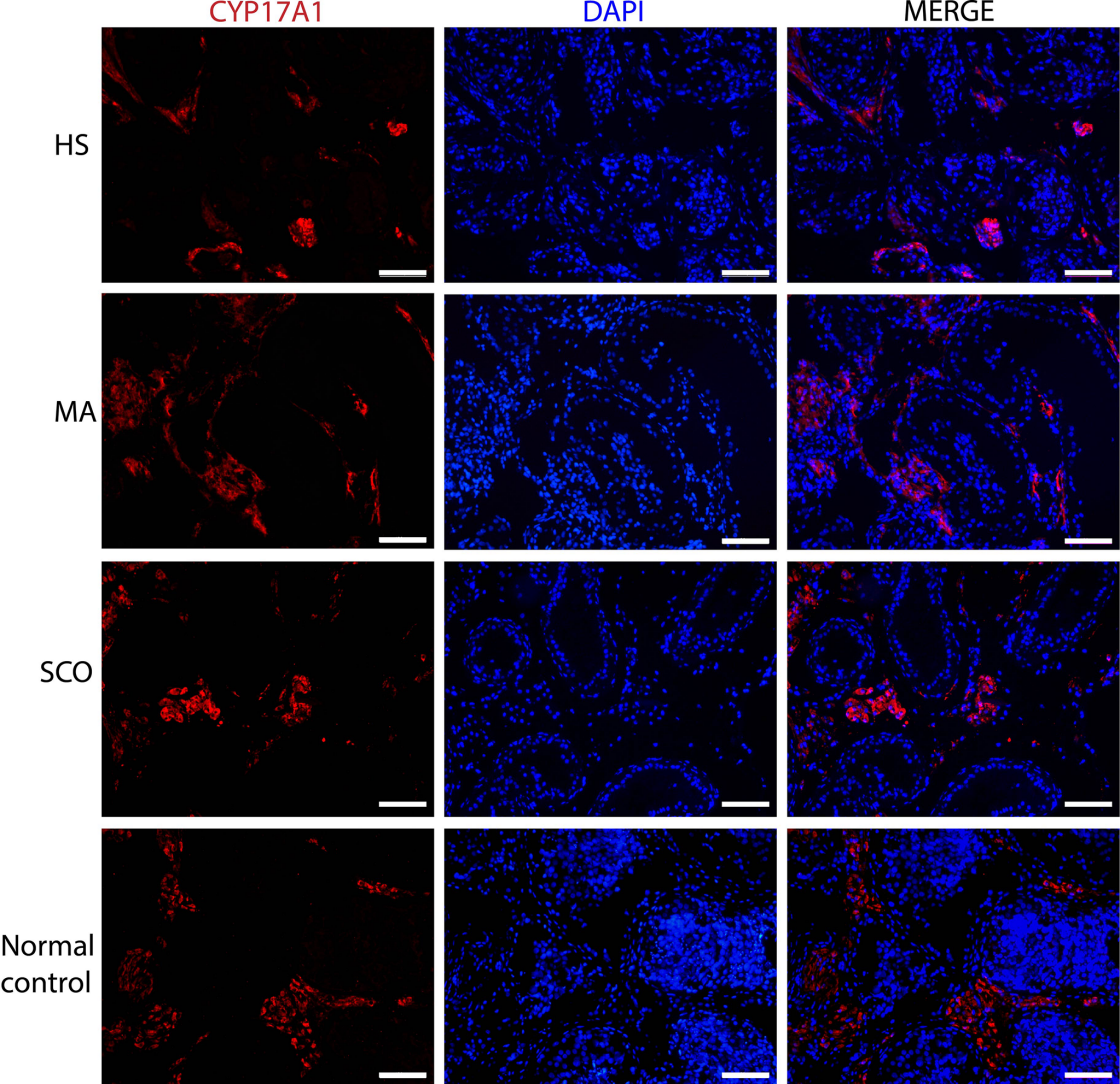
Immunofluorescence staining of Leydig cell marker in un-dilated seminiferous tubules from HS, MA, SCO, and NC samples. CYP17A1 (red) for Leydig cells, DAPI (blue) for nuclear staining. Scale bar: 100 μm.

Leydig cells visualized *via* CYP17A1 expression showed no difference in distribution between any of the NOA samples and the normal controls (**Figure 3**). Due to the tiny testis biopsies the interstitial tissues were not fixed well and quantification of the CYP17A1-positive Leydig cells was not done in this study.

#### *γH2AX*-Positive Cells

**Figure 4:**
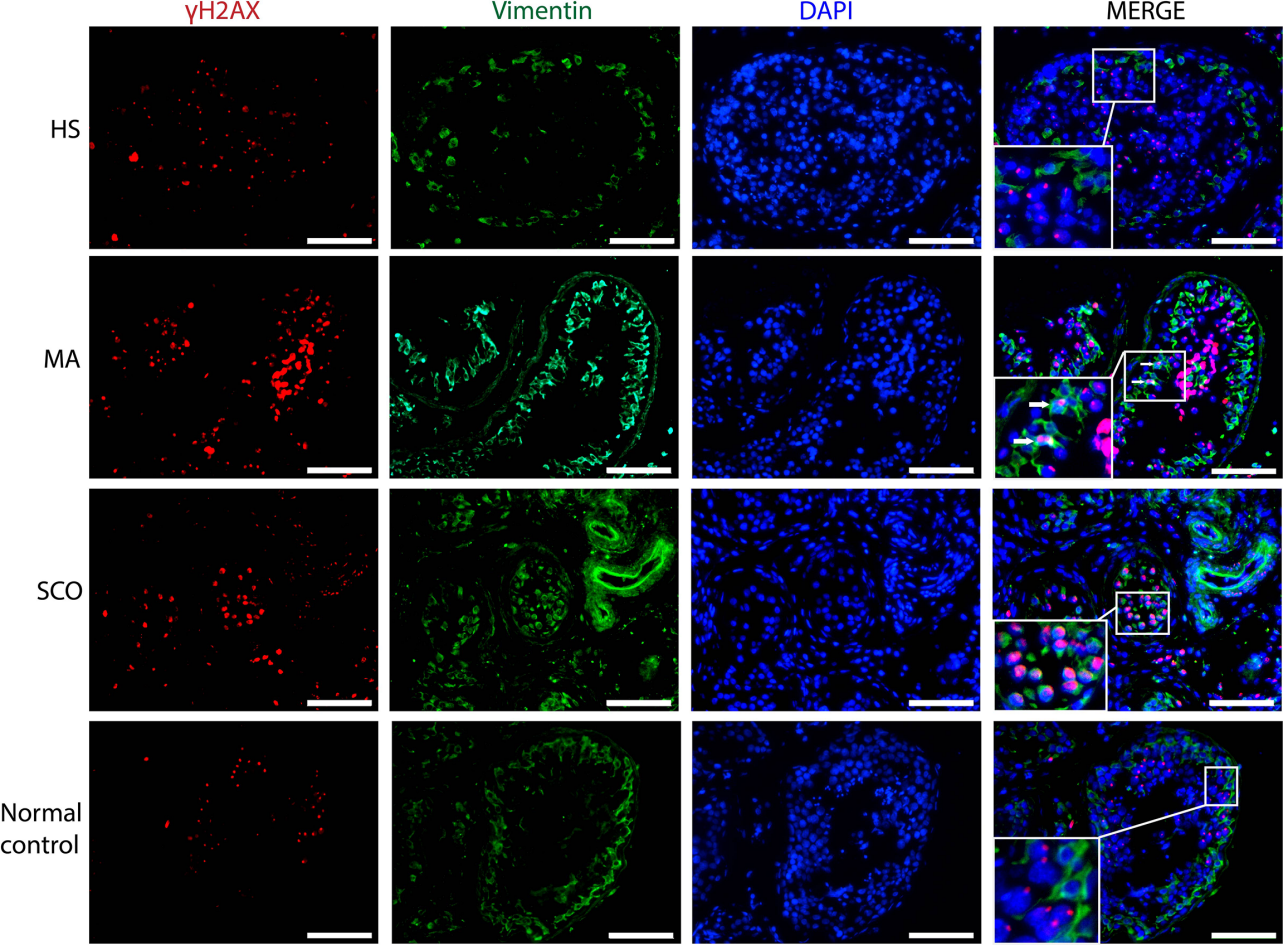
Immunofluorescence staining of γH2AX (red), somatic cell marker Vimentin (green), nuclear marker DAPI (blue) in un-dilated seminiferous tubules in samples from HS, MA, and SCO samples. White arrow indicated γH2AX-positive Sertoli cells in MA. As almost all Sertoli cells expressed γH2AX in SCO, we did not add arrow to indicate them. There were no γH2AX-positive Sertoli cells in HS and normal control group. Scale bar: 100 μm.

In HS, there was a strong homogenous nuclear staining and prominent γH2AX foci in most of the germ cells (**Figure 4**). Some nuclear staining was weak and dispersed, but most were dotted and strong. Almost no γH2AX expression was detected in Sertoli cells stained with Vimentin. In MA, γH2AX was strongly expressed in the nuclei of germ cells in a dotted and dispersed pattern. There were a few Sertoli cells with γH2AX staining (**Figure 4**). In SCO, almost all Sertoli cells showed a pronounced expression γH2AX in the nuclei (**Figure 4**).

#### Germ Cells

**Figure 5:**
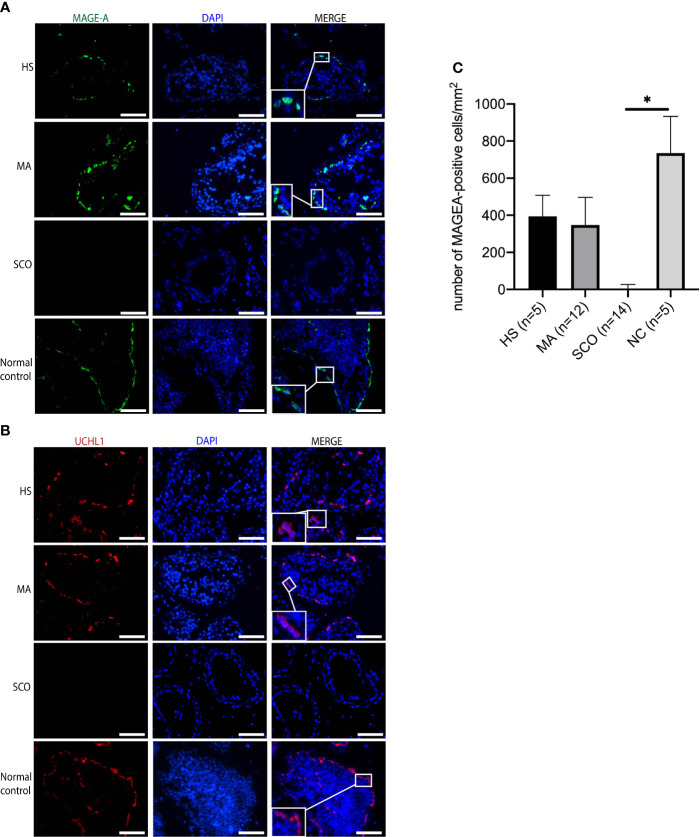
Immunofluorescence staining of germ cell markers in un-dilated seminiferous tubules from HS, MA, SCO, and NC samples. **(A)** MAGE-A (green) for germ cells, **(B)** UCHL1 (red) for germ cells, DAPI (blue) for nuclear staining, Scale bar: 100 μm. **(C)** The number of MAGE-A-positive cells per mm^2^ was counted based on ten tubules of each biopsy. Asterisk indicated significant difference between SCO and normal control group (*p < 0.05).

Both germ cell markers, MAGE-A and UCHL1, were positively expressed in the un-dilated seminiferous tubules of HS and MA patients indicating the presence of germ cells (**Figures 5A, B** **)**. The average number of MAGE-A-positive cells per tubule was 17 in HS, 15 in MA, 0 in SCO, and 26 in normal control group. The number of MAGE-A-positive cells per mm^2^ was not significantly different in HS and MA compared to the normal group (**Figure 5C**). In contrast, no expression of MAGE-A and UCHL1 was present in SCO patients (**Figures 5A-C** **)**. The staining patterns in these three types were mainly cytoplasmic and the location of positive cells was near the basement membrane of the seminiferous tubules as observed in the normal control.

### qPCR Analysis of Niche Related Cells

**Figure 6:**
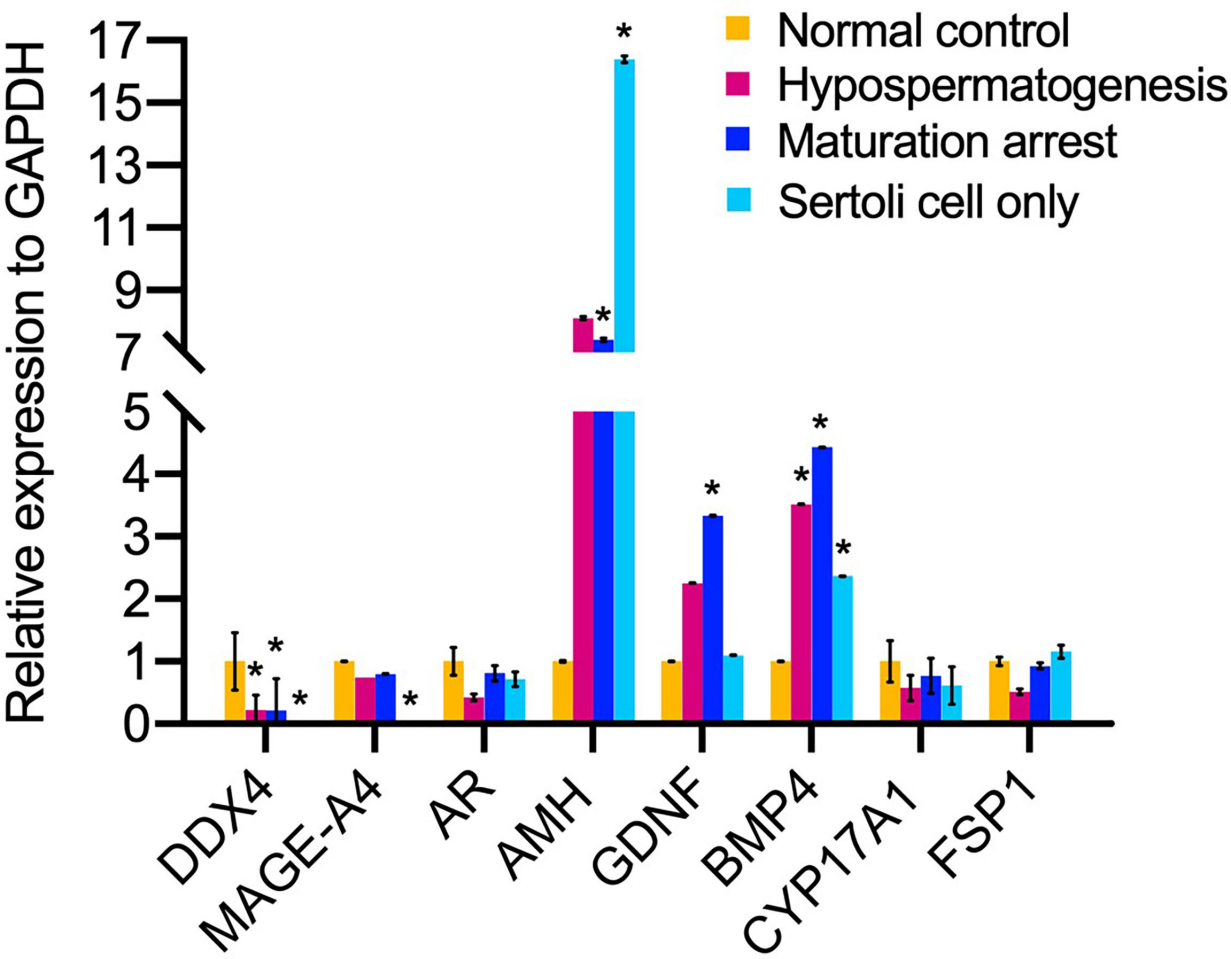
The mRNA expression analysis of niche related cells in un-dilated seminiferous tubules from non-obstructive azoospermia patients (NOA) and healthy adult men. The relative mRNA expression of germ cell *DDX4*, *MAGE-A4*, and somatic cell *AMH*, *AR*, *GDNF, BMP4*, *CYP17A1*, fibroblast-specific protein 1 (*FSP1*) to internal control *GAPDH*. Kruskal-Wallis test, *p < 0.05.

The mRNA expression of DEAD-box helicase 4 (*DDX4*, also named *VASA*) germ cell-specific gene was significantly decreased in HS, MA, SCO compared to normal control group (*P*=0.006, *P*=0.004, *P*=0.004, respectively) (**Figure 6**). In SCO, the mRNA expression of *MAGE-A4* was significantly downregulated (*P*=0.004) (**Figure 6**). The mRNA expression of *AMH* (indicating immature Sertoli cells) was significantly increased in MA and SCO compared to normal control group (*P*=0.001, *P* =0.0002, respectively) (**Figure 6**). Contrary to the *AMH*, the mRNA expression of androgen receptor (*AR*) (indicating mature Sertoli cell) showed no difference in the three types of NOA samples compared to the normal group (**Figure 6**). The mRNA expression of GDNF was significantly increased in MA (*P*=0.0003) (**Figure 6**). The mRNA expression of BMP4 showed a significant increase in HS, MA, and SCO (*P*=0.02, *P*=0.0005, *P*=0.02, respectively) (**Figure 6**).

### Correlation Between mRNA Expression and Serum FSH

In MA, serum FSH showed a significant negative correlation with mRNA expression of *CYP17A1* (r=-0.55, *P*=0.04) (**Table 2**). No other correlations were found between the remaining genes and hormone values.

**Table 2:** Correlation between mRNA expression and clinical hormones.

	HS (n = 5)					
Variable	AR	AMH	GDNF	BMP4	FSH	Inhibin B
VASA	-0.30,.68	0.40,.52	0.20,.78	-0.10,.95	0.10,.95	0.50,.45
MAGE-A4	-0.30,.68	-0.90,.08	-0.70,.23	0.00, >0.99	-0.10,.95	-0.10,.95
CYP17A1	-0.50,.45	-0.10,.95	-0.70,.23	-0.90,.08	0.10,.95	-0.40,.52
FSP	0.10,.95	0.30,.68	0.10,.95	-0.70,.23	0.70,.23	-0.20,.78
LH	0.67,.27	0.67,.27	0.36,.63	-0.56,.37	0.36,.63	-0.87,.07
T	0.60,.35	0.80,.13	0.40,.52	-0.50,.45	0.20,.78	-0.80,.13
	**MA (n=14)**					
**Variable**	**AR**	**AMH**	**GDNF**	**BMP4**	**FSH**	**Inhibin B**
VASA	-0.02,.95	0.02,.93	0.22,.46	0.20,.48	0.41,.15	-0.08,.80
MAGE-A4	-0.29,.33	0.09,.76	0.18,.56	0.01,.99	0.49,.09	-0.27,.36
CYP17A1	0.45,.11	-0.13,.65	0.09,.75	0.13,.67	-0.55,.04	0.26,.36
FSP	0.52,.06	0.03,.92	0.28,.34	0.20,.50	-0.41,.14	0.45,.11
LH	-0.25,.38	0.17,.55	-0.46,.10	-0.34,.23	0.58,.03	-0.68,.01
T	0.36,.20	0.02,.94	0.28,.32	0.29,.32	-0.11,.71	0.15, 61
	**SCO (n=15)**					
**Variable**	**AR**	**AMH**	**GDNF**	**BMP4**	**FSH**	**Inhibin B**
VASA	-0.29,.29	-0.18,.52	0.35,.30	0.23,.50	-0.28,.30	-0.07,.80
MAGE-A4	0.02,.95	-0.02,.95	-0.40,.75	0.60,.42	-0.23,.41	-0.32,.24
CYP17A1	-0.19,.49	0.01,.96	0.12,.68	0.31,.25	-0.36,.19	0.30,.28
FSP	0.03,.91	-0.10,.71	0.13,.65	0.08,.78	-0.06,.82	0.18,.51
LH	0.25,.37	0.20,.47	-0.13,.64	-0.09,.75	0.66,.01	-0.31,.27
T	-0.21,.45	0.07,.80	0.15,.59	-0.07,.81	0.40,.14	0.31,.27

Values are r (correlation coefficient), P value. P <.05 was considered significant by Spearman’s rank correlation.

## Discussion

This study demonstrated pronounced testicular differences within the un-dilated seminiferous tubules between different types of NOA patients and normal control group. The maturation state of Sertoli cells, the number of germ cells, and the thickness of tubule wall were distinct in NOA subtypes. Collectively, the microenvironments within the un-dilated tubules are different in different subtypes of NOA patients.

The maturation state of Sertoli cells was evaluated by AMH and AR expression on both mRNA and protein level. The higher *AMH* mRNA expression and more tubules with AMH expression in all three NOA subtypes compared to that in the normal group suggests that there were more immature Sertoli cells within the un-dilated tubules from all three NOA subtypes. Earlier studies also showed that immature Sertoli cells were observed in the testis from infertile adult men (32–34). After a higher dilution of AMH antibody employed, we found that there was a threshold level of detection and variable AMH expression in the normal control group. The results are consistent with a previous report that showed both AMH positive staining within the seminiferous tubules of patients with Sertoli-cell-only syndrome (SCOS) and in men with normal spermatogenesis, but staining intensity was stronger in SCOS than in normal group (28). Furthermore, in adult men, AMH is secreted in both serum and seminal plasma (35). It was reported that the seminal AMH concentration was variable ranging from undetectable to a high level (36) suggesting a Sertoli cell secretion. Collectively, this argues for a mild AMH expression in tubules from normal fertile men. In HS and MA, the number of AR-positive mature Sertoli cells appeared to be reduced. This tendency was also shown in *AR* mRNA expression level. In SCO, the number of SOX9- and AR-positive cells was slightly higher than the normal group, but no significant differences were found, and the *AR* mRNA expression showed a decreased tendency. Thus, there are both mature and immature Sertoli cells in SCO patients, to what extend both were increased needs to be addressed in a future study. The increased expression of AMH in Sertoli cells may reflect a maturation failure of Sertoli cells in connection with puberty (37, 38) or alternatively de-differentiation of mature Sertoli cells to acquire a more immature state (34). However, the current study is unable to distinguish between maturation failure and de-differentiation of the Sertoli cells.

It has been reported in mice that the overexpression of GDNF showed accumulation of undifferentiated spermatogonia (14) and inhibiting GDNF signaling could promote differentiation of SSC (39). We found GDNF mRNA overexpressing in MA suggesting GDNF could contribute to the maturation failure in MA. It has been reported that FSH induces the GDNF expression (40) and the higher FSH values (i.e. 20.6 IU/L in MA NOA subgroup as compared to 1.5-12.4 IU/L in the normal group) could explain the observed higher expression of GDNF. All three subtypes of NOA showed overexpression of BMP4 mRNA confirming a previous study that showed BMP4 overexpression in MA-NOA and SCO-NOA at the protein level in relation to control group (41). In contrast, mRNA expression of BMP4 was reported lower in SCO (42). This discrepancy may be due to mixing the control group with both tissues from men with hypospermatogenesis and normal spermatogenesis.

The germ cell status was evaluated by using qPCR and immunofluorescence. The mRNA expression of *DDX4* and *MAGE-A4* was significantly reduced in SCO and exhibited a decreased tendency in HS and MA. This is consistent with other studies that reported a reduced germ-cell niche in HS and MA from infertile men (43–45). The number of MAGE-A-positive germ cells was slightly reduced in HS and MA while absent in SCO. Similarly, the germ cell specific UCHL1 expression was absent in SCO. The attenuated germ cell numbers may either be related to meiotic defects (46, 47) and/or impairment and immaturity of Sertoli cells being unable to support full germ cell maturation.

The presence of histone H2AX phosphorylation (γH2AX) was used to identify germ cells in the prophase of the first meiotic division (48), but Sertoli cells expressing DNA damage response also become stained (25). H2AX is a histone variant that belongs to the H2A family and prevent genome instability and cancer (49–51), while the phosphorylated form γH2AX is regarded as a robust marker of DNA double-strand breaks (DSBs) (52, 53). We found that the γH2AX expression was different between three types of NOA patients. There were more Sertoli cells expressing γH2AX in SCO than in HS and MA biopsies. The accumulation of γH2AX positive staining in Sertoli cells demonstrated that the Sertoli cells may be undergoing accelerated degradation.

The present study suggested that Sertoli cells in un-dilated seminiferous tubules of NOA patients were immature and expressed an increased DNA damage compared to normal controls. In MA, few Sertoli cells appeared to have DNA damages response. In SCO, many γH2AX-positive Sertoli cells may undergo DNA damage response.

Collectively, the present study suggests that un-dilated seminiferous tubules from three subtypes of NOA patients show a different expression of cell specific markers that most likely reflect their compromised ability to sustain spermatogenesis or alternatively that compromised germ cells influence Sertoli cell function. However, both HS and MA subtype of NOA patients demonstrate, in un-dilated seminiferous tubules, the quantitative presence of germ cell numbers approaches that of normal testis in some instances. It may therefore be envisioned that these germ cells could be matured to haploid germ cells and used in connection with ART. This will require the development of an *in vitro* culture system providing a proper environment, for instance by co-culture with spent media from cultures of mature normal Sertoli cells. Alternatively, testis tissue from men with NOA could be cultured together with mature Sertoli cells from a normal testis without direct cell contact between the NOA tissue and the supplied normal Sertoli cells. Alternatively, or in combination, growth factors and hormones known to advance meiosis may be used to advance meiosis in cultures of testis tissue from NOA patients in whom sperm retrieval was unsuccessful (54, 55). The fact that some of these men with NOA actually present with a few dilated areas of seminiferous tubules with spermatogenesis suggests that it is possible to define conditions of sufficient quality to advance meiosis to the haploid state.

The thicker tubule wall of seminiferous tubules in NOA patients with SCO may affect the contractility of the tubules and the propulsion of the tubular contents to the rete testis.

A limitation of our study is the relatively small sample size. More samples are necessary in the future for further exploration of mechanisms behind NOA.

In conclusion, this study provides insights into understanding the un-dilated (atrophic) tubules which constitute a major part of seminiferous tubules of NOA patients. Improvement of Sertoli cell function either during *in vitro* culture or by co-culture with Sertoli cells from fertile men may constitute strategies for fertility restoration in patients with different types of NOA that fail surgical sperm retrieval. The impairment and immaturity of Sertoli cells and germ-cell loss are likely to contribute to the impaired spermatogenesis.

## Data Availability Statement

The original contributions presented in the study are included in the article/**Supplementary Material**. Further inquiries can be directed to the corresponding author.

## Ethics Statement

The studies involving human participants were reviewed and approved by the Regional Ethical committee of the Capital Region of Denmark (H-16033784) and the Region of Southern Denmark (S-20200088). The patients/participants provided their written informed consent to participate in this study.

## Author Contributions

CJ, DW, LM, AG, NJ, MF, DO, LD, JF, CA, JS conceived and designed the experiments. CJ collected testis biopsies for experimental use. DW performed the experiments. CJ, DW, LM, SH, SP, EN, CA performed data analysis and interpretation. DW wrote the manuscript. All authors contributed to the article and approved the submitted version.

## Funding

This article is part of the ReproUnion collaborative study, co-financed by EU Interreg ÖKS, Capital Region of Denmark, Region Skåne and Ferring Pharmaceuticals. Further, the support from Vissing Fonden (519140 AHO/PPT) and the Danish Child Cancer Foundation (2021-7395) is greatly acknowledged.

## Conflict of Interest

The authors declare that the research was conducted in the absence of any commercial or financial relationships that could be construed as a potential conflict of interest.

## Publisher’s Note

All claims expressed in this article are solely those of the authors and do not necessarily represent those of their affiliated organizations, or those of the publisher, the editors and the reviewers. Any product that may be evaluated in this article, or claim that may be made by its manufacturer, is not guaranteed or endorsed by the publisher.
