# Supplementary material for: Sertoli and Germ Cells Within Atrophic Seminiferous Tubules of Men With Non-Obstructive Azoospermia
Source: Front Endocrinol (Lausanne). 2022 Jun 2;13:825904. doi: 10.3389/fendo.2022.825904 (PMC9201000; doi:10.3389/fendo.2022.825904)
Supplement: Supplementary file 1 [file Table_1.docx]

**Supplementary Table 1** Primary antibodies and target cells

| Antibody | Target cell | Concentration | Catalog number | Manufacturer | Reference |
| --- | --- | --- | --- | --- | --- |
| MAGE-A | spermatogonia and spermatocyte | 1:100 | sc-20034 | Santa Cruz | 23 |
| UCHL1 | spermatogonia | 1:100 | sc-271639 | Santa Cruz | 24 |
| γH2AX | germ cell and somatic cell | 1:1500 | NB100-384 | Novus Biologicals | 26 |
| Vimentin | somatic cell | 1:200 | sc-6260 | Santa Cruz | 27 |
| SOX9 | Sertoli cell | 1:100 | AB5535 | Merck Millipore | 28 |
| AMH | immature Sertoli cell | 1:100 | AF2748 | R&D systems | 28 |
| AR | mature Sertoli cell | 1:100 | ab108341 | abcam | 29 |
| ACTA | peritubular myoid cell | 1:150 | ab5694 | abcam | 30 |
| CYP17A1 | Leydig cell | 1:200 | sc-46084 | Santa Cruz | 23 |

| Genes | Product No. | Company |
| --- | --- | --- |
| *GAPDH* | Hs02786624_g1 | Thermo Fisher Scientific |
| *MAGE-A4* | Hs01025554­_m1 | Thermo Fisher Scientific |
| *DDX4* | Hs00987119_g1 | Thermo Fisher Scientific |
| *CYP17A1* | Hs01124136_m1 | Thermo Fisher Scientific |
| *AMH* | Hs00174915_m1 | Thermo Fisher Scientific |
| *AR* | Hs00171172_m1 | Thermo Fisher Scientific |
| *FSP1* | Hs00210845_m1 | Thermo Fisher Scientific |
| *GDNF* | Hs01931883_s1 | Thermo Fisher Scientific |
| *BMP4* | Hs03676628_s1 | Thermo Fisher Scientific |

**Supplementary Table 2** PCR Taqman primer assays
